# BreastWatch: A Varian Eclipse script tool for Community‐Based automatic evaluation of breast treatment plans

**DOI:** 10.1002/acm2.70080

**Published:** 2025-04-01

**Authors:** Stefano Agostinelli, Daniele Zefiro, Stefania Garelli, Francesca Cavagnetto, Marco Gusinu, Monica Bevegni, Gladys Blandino, Alessandra Fozza, Fabrizio Levrero

**Affiliations:** ^1^ UO Fisica Sanitaria IRCCS Ospedale Policlinico San Martino Genova Italy; ^2^ UO Radioterapia Oncologica IRCCS Ospedale Policlinico San Martino Genova Italy

**Keywords:** automatic evaluation, breast radiation therapy, eclipse, scripting, treatment plan scoring

## Abstract

**Purpose:**

This work introduces BreastWatch, a Varian Eclipse script tool designed to help medical physicists, dosimetrists, and radiation oncologists easily inspect and improve External Beam Breast Treatment (EBBT) plans using automatic evaluation of protocol dose‐constraints enhanced by a Community‐Based approach.

**Methods:**

BreastWatch examines Eclipse EBBT Plans and Plan Sums, automatically testing the plan's DVH against built‐in international EBBT protocol (NRG RTOG1005, ICR Fast‐Forward, ICR FAST, and EUROPA 1.2) dose‐constraints. Results are displayed using traffic lights and an overall BW‐SCORE. To overcome protocol dose‐constraints limitations, the script can export plan data and dose values in CSV format to build a Community‐Based Plan Library. These data are automatically read back in BreastWatch to check plans against the statistical distribution of similar Community plans and compute a CB‐SCORE, which complements the protocol‐based BW‐SCORE.

In this paper, BreastWatch v.1.6.5.0 script's design and strengths are examined and discussed, after extensive testing with more than 800 EBBT plans by six experienced planners. In a preliminary analysis, the usefulness of the CB approach is investigated by examining the chronological series of two dose‐constraints values (Contra‐Lung V3Gy and Heart Average Dose) before and after its introduction in BreastWatch.

**Results:**

We found BreastWatch to speed up the EBBT planning process, while at the same time helping to improve the treatment plans in terms of PTVs coverage and uniformity, sparing of OARs, consistency, and robustness among different planners. The preliminary analysis of the chronological series of two dose‐constraints values for a subgroup of 68 EBBT plans shows a trend (*p*‐value = 0.07) for values to be improved and more consistent after the CB approach introduction.

**Conclusions:**

The BreastWatch script is a useful tool for the automatic evaluation of Eclipse EBBT plans. BreastWatch and its Community‐Based features have been found to be a simple but effective automation approach to improve the EBBT planning process.

## 1 INTRODUCTION

Given the large number of patients treated with radiotherapy for breast cancer, External Beam Breast Treatment (EBBT) planning is one of the most time‐consuming tasks of every Medical Physics Unit working for Radiation Oncology. Nowadays, EBBT plans can involve different treatment techniques including 3DCRT, IMRT, VMAT/RA, a Hybrid combination,^1^ Deep Inspiration Breath Hold (DIBH) and skin bolus. Different prescription schemas are available including Partial Breast Irradiation (PBI) and moderate or ultra hypo‐fractionation, which are now commonly used for a vast selection of patients according to international consensus and treatment protocols/trials like NRG RTOG1005, IRC Fast‐Forward, IRC FAST and EUROPA.^2^, ^3^, ^4^, ^5^ Each of these protocols contain specific dose‐constraints for PTVs and OARs, so EBBT planners and plan reviewers have to deal with the selection of the correct clinical goals and a plethora of different dose‐constraints. In addition, protocol dose‐constraints are generic, treatment technique‐dependent, not considering center‐specific experience and contouring variations, in general, not sufficient to check the real *quality* of an EBBT plan. For all these reasons, in clinical practice, there is a strong need of tools that can help the planners to speed up the plan evaluation and to achieve plan *quality* metrics that not only satisfy protocol dose‐constraints, but are optimized for the single patient, treatment technique and center‐specific aspects.

Automation methods offer a natural solution to help deal with the increasing complexity of EBBT planning and its optimization for modern treatment techniques. Knowledge‐based (KB), machine learning (ML), or deep learning (DL) artificial intelligence (AI) algorithms have been recently proposed and validated for the EBBT planning process.^6^, ^7^, ^8^, ^9^, ^10^, ^11^, ^12^ The BreastWatch script introduces a simple automatic evaluation method, complementary to AI techniques, for the analysis and the improvement of EBBT plans by automated comparison with breast treatment protocol dose‐constraints and Community‐Based (CB) data accumulated by the local Community of script's users.

## 2 METHODS

### 2.1 Overview of the BreastWatch script

BreastWatch is a plugin script for the Varian Eclipse Treatment Planning System. BreastWatch was developed for Eclipse v.13.5 using Varian Eclipse Scripting API (ESAPI) and Microsoft Visual Studio C# Community 2019. The following text refers to BreastWatch v.1.6.5.0 for Varian Eclipse v.13.5. BreastWatch v.1.7.5.0 for Eclipse v.18 is also available with similar features.

**FIGURE 1:**
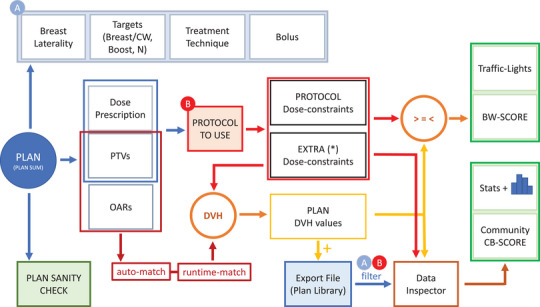
Workflow of the BreastWatch script. At the start, BreastWatch performs Plan Sanity Check and finds out plan properties (A‐box): Laterality, Targets, Treatment Technique, and Bolus. Laterality can be LEFT, RIGHT, or BILATERAL. Targets specify if the plan treats also BOOST and N/Lymph‐nodes targets. The main PTV (Breast) accepts modifiers CHESTWALL, EXP, and EXP_HS to specify a Chest‐Wall target with/without expander/implant and implant‐sparing contouring.^16^ Treatment Techniques can be 3DCRT, IMRT, VMAT, and HYBRID and can specify modifiers BH (Breath‐Hold), AP (Autoplanned) and TANG (Tangential). Bolus can be NONE, ALL, or PARTIAL. After initial setup, the protocol to use is selected (B), and the DVH of matched PTVS and OARs is calculated. Plan DVH values are compared with protocol dose‐constraints (standard + extra) to compute traffic lights and the BW‐SCORE and can be appended to the Export File, which serves as a Community Plan Library. Using Data Inspector, each plan DVH value is compared to the values for similar plans found in the Community Plan Library, and the CB‐SCORE is calculated. Similar plans are fetched in the Library by filtering on protocol (B) and A‐box properties.

The BreastWatch script is a tool for the automatic evaluation of Eclipse EBBT plans. The complete workflow of the BreastWatch script is presented in Figure 1. Basically, the script:

1. automatically selects the correct EBBT protocol to use for the plan,
2. computes the protocol‐specific dose‐constraints values by looking at the plan's DHV for matched PTVs and OARs,
3. shows dose‐constraints results with traffic lights and computes a global BreastWatch‐SCORE, and
4. exports the plan data and dose‐constraints results in a local CSV Export File, which can serve as a Community Plan Library to be examined offline.

Since BreastWatch v.1.5, the Export File is automatically read back by the script and is used to show the plan dose‐constraints values compared to appropriate Community Plan Library data and compute a Community‐Based CB‐SCORE. BreastWatch's traffic lights, BW‐SCORE, and CB‐SCORE can help the planner to speed up and improve the EBBT planning process.

### 2.2 Initialization and protocol selection

The script is launched from the Scripts menu of the Eclipse External Beam module and analyzes the in‐context EBBT Plan or Plan Sum. BreastWatch natively supports 3DCRT, IMRT, and VMAT/RA Eclipse EBBT plans but also works with Dose Plans (plans containing only dose calculation, but no treatment beams), so it's possible to examine plans imported in Eclipse from other Treatment Planning Systems or special‐platform plans like Tomotherapy plans.

At the start, BreastWatch examines the Plan/Plan Sum and checks the dose prescription and number of PTVs to automatically select the correct EBBT protocol to apply. Supported protocols include NRG RTOG1005, ICR Fast‐Forward, ICR FAST and EUROPA Trial PBI/WBI 1.2.^2^, ^3^, ^4^, ^5^ The script then inspects the Plan to find out the Treatment Technique (3DCRT, IMRT, VMAT/RA, Hybrid, and Breath‐Hold), the breast Laterality (LEFT, RIGHT, or BILATERAL), the presence of a Bolus, the in‐use PTVs (Breast/Chest Wall, Boost, and N/Lymph‐nodes) and OARs (Ipsilateral Lung, Contralateral Lung, Heart, Left Coronary, Contralateral Breast, Esophagus, Stomach, Liver, Thyroid, and Skin). Matching of PTVs and OARs is automatic and based on structure names, but can be corrected at runtime by the user. In addition, BreastWatch performs a Plan Sanity Check (PSC) of the Plan/Plan Sum by examining the number of isocenters, total MUs, beams dose‐rate, jaws width for IMRT/VMAT plans, and couch rotation for setup beams.

After initialization and PSC, BreastWatch analyzes the plan's DVH and automatically checks the protocol‐specific dose‐constraints. To overcome standard protocol dose‐constraints limitations, BreastWatch uses extended (standard + extra) protocol dose‐constraints to better ascertain the *quality* of the EBBT plan. A complete list of protocols and extra dose‐constraints is available in the script's documentation.

### 2.3 BreastWatch User Interface

**FIGURE 2:**
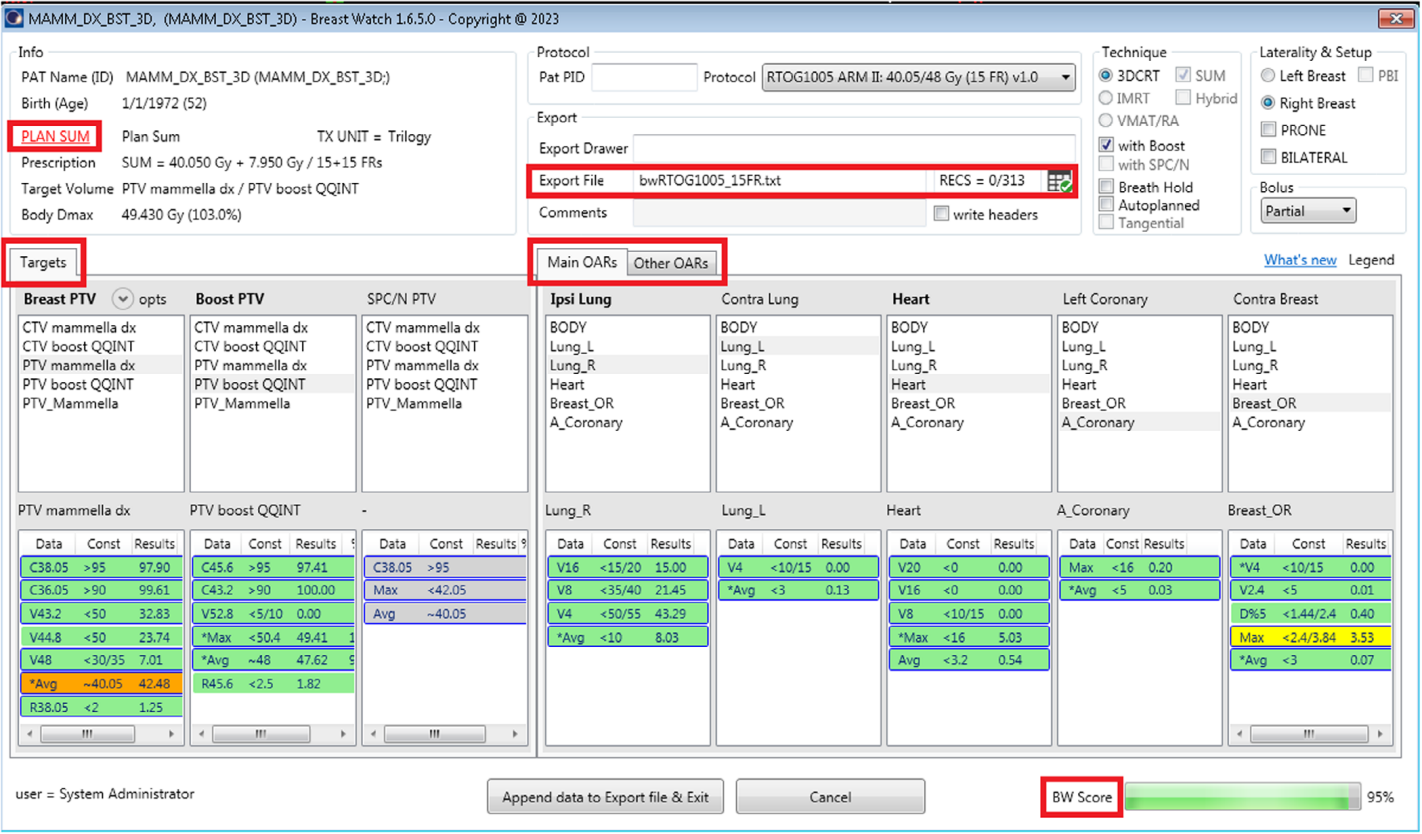
BreastWatch main window User Interface. Panels in the upper part show patient, plan, data export, and protocol information. The PLAN/PLAN SUM label displays the result of the PSC (green = all tests passed, red = some test not passed). Export File specifies the Community Plan Library CSV file to which plan data can be exported. RECS reports the number of records found in the Community Plan Library (current user records/total records). The icon next to RECS signals if the current user can access the whole library or only his/her own records. In the Targets and Main/Other OARs panels, the Listviews (first row) allow associating plan structures to Targets and OARs volumes and (second row) show DVH dose‐constraints values with traffic light colors. The BW‐SCORE bar shows the percent of (extended) protocol dose‐constraints fulfilled by the plan.

**FIGURE 3:**
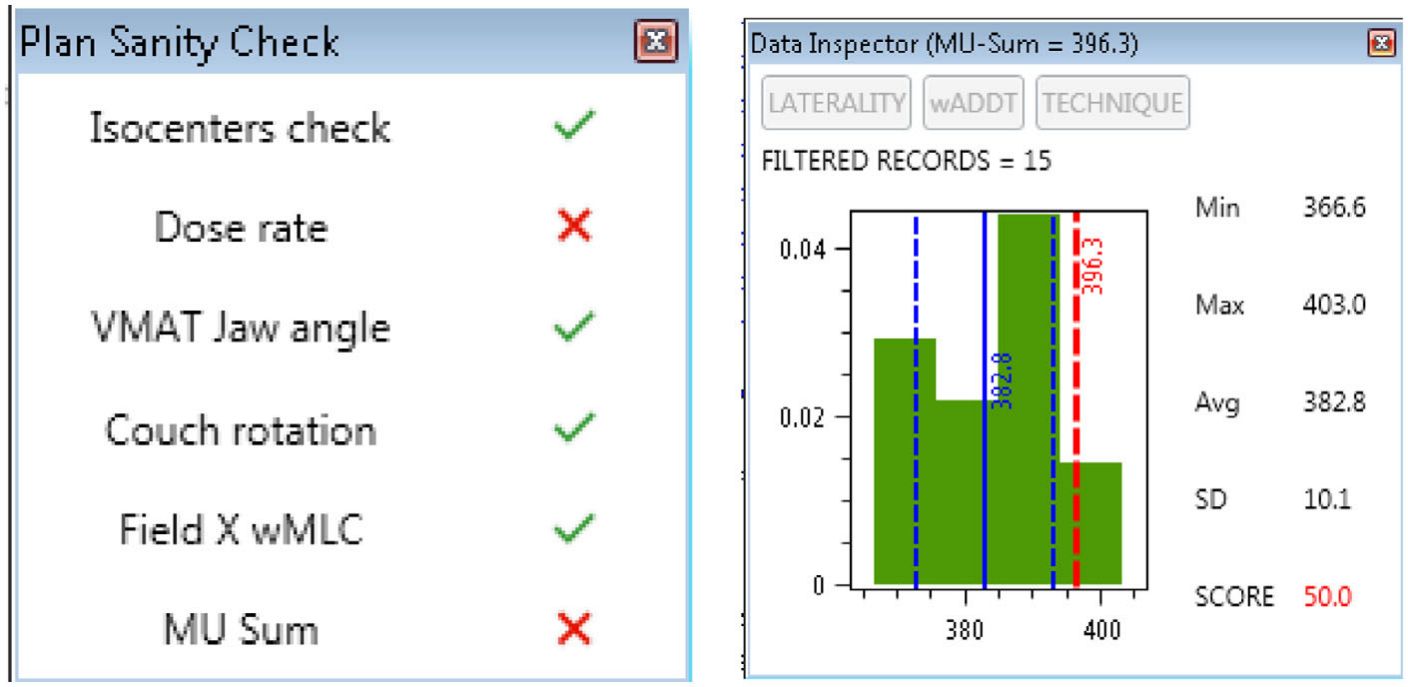
Left: Plan Sanity Check window User Interface. Double‐clicking on the MU‐Sum icon opens a Data Inspector window (Right) for MU‐Sum comparison with Community data. In this example, the MU‐Sum = 396.3 (red bar) is above the Community MU‐Sum average = 382.8 (solid blue bar) and average + 1SD (dashed bar).

The script main window (Figure 2), on the top, shows five panels with information about the patient, plan, data export, and the protocol selected for analysis. The PLAN/PLAN SUM label in the Info panel is linked to the PSC and signals with green/red colors if all plan checks were passed or not. A double‐click on the label opens the PSC window (Figure 3). Protocol‐specific and extra dose‐constraints are organized in Listviews using traffic light colors (green‐yellow‐red) for each PTV and OAR. The yellow color is used only for constraints specifying optimal/mandatory values. Extra dose‐constraints are displayed using a star (*) and green‐pale yellow‐orange colors. OARs are grouped in two panels: “Main OARs” and “Other OARs.” The former includes the Ipsilateral Lung, Contralateral Lung, Heart, Left Coronary, and Contralateral Breast, while the latter includes Esophagus, Stomach, Liver, Thyroid, and Skin. On the bottom right of the main window, the BreastWatch‐SCORE (BW‐SCORE) bar summarizes the percent of fulfilled PTVs and Main OARs dose‐constraints (0% = no constraint satisfied, 100% = all constraints satisfied).

### 2.4 Community Plan Library

**FIGURE 4:**
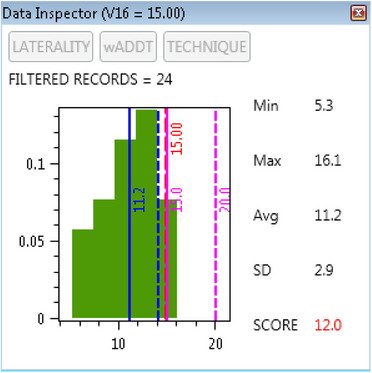
BreastWatch Data Inspector UI for Lung‐V16 dose‐constraint. In this example, 24 “similar” records (plans) have been found in the Community Plan Library. The plan V16 value is 15 (red bar); while this value it's below the protocol dose‐constraint V16 ≤ 20 (purple bar), it lies above the Community mean (solid blue bar) + 1 SD (dashed blue bar). Inspector's Community SCORE is just 12 because only 12% of the 24 Community plans have greater (worse) V16 values.

Using BreastWatch, planners can save plan and dose‐constraints data in the CSV Export File specified in the Export Drawer and Export File Name boxes. The Export Drawer is configured through the script's Preferences text file. The Export File can be examined offline as a detailed Community Plan Library to examine treatment statistics of saved data for the local Community of script's users. Since version 1.5, BreastWatch offers a built‐in *Data Inspector* feature, which automatically reads back the Export File to compute for each dose‐constraint the statistical distribution of dose‐constraints values for Community plans. Double‐clicking a dose‐constraint in a Listview, the Data Inspector window opens (see Figure 4), showing the plan dose‐constraint value and protocol dose‐constraint superimposed to the histogram of values for Community plans with an indication of the statistical minimum, maximum, mean, and standard deviation. During this process, the Community Plan Library is filtered by protocol, Laterality, type of PTVs, and Treatment Technique to provide an accurate snapshot of appropriate Community data. In this way, the examined plan is compared only to the cohort of Community plans with the same characteristics. The Community‐Based SCORE (CB‐SCORE = % of “worse” Community plans) is also computed, providing evidence if the plan is “compatible” with (or even better than) Community data or not. Access to the Community Library in BreastWatch is granted through a privilege key set in the script's Preferences text file, which can allow users to see all data or only data saved by the currently logged user.

### 2.5 BreastWatch in the planning practice

BreastWatch has been extensively tested with more than 800 EBBT plans by six experienced planners. Most of these plans were following NRG RTOG1005 fractionation (50% of the plans) and ICR Fast‐Forward fractionation (45% of the plans). 3DCRT, VMAT/RA, and Hybrid Plans or Plan Sums were all considered. The testing was performed mainly on Varian Eclipse v.13.5 and more recently, on Eclipse v.18 using Clinical Goals. After conventional EBBT planning, the planners and the plan reviewers were asked to use BreastWatch as a tool for plan evaluation and improvement. This involved visual inspection of traffic lights, BW‐SCOREs, and Community‐SCOREs. In the end, the general impressions of BreastWatch effectiveness in plan analysis and optimization were gathered.

### 2.6 Preliminary analysis of the Community‐Based tool impact

To evaluate the potential impact of the Community‐Based Data Inspector tool on planning, we analyzed constraint data results before and after its introduction in BreastWatch v1.5. This preliminary analysis focused on two parameters contained in the ICR Fast‐Forward plan dataset: Contra‐Lung V3Gy (%) and Heart Average Dose (Gy). In BreastWatch ICR Fast‐Forward protocol, the Contra‐Lung V3Gy and the Heart Average Dose are extra dose‐constraints suggested to be ≤10% and ≤2 Gy, respectively. The two parameters were analyzed for RIGHT breast treatment VMAT plans with/without Boost PTV and no DIBH. The choice of the parameters and laterality was made to detect subtle variation in planners behavior for dose‐constraints that are secondary (Contra‐Lung V3Gy) or often difficult to achieve using VMAT (Heart Average Dose). The analysis examined the change detection, BEFORE (34 records) and AFTER (34 records) Data Inspector introduction, of the chronological series of the aforementioned parameters and their quadratic deviations from the mean using non‐parametric Mann‐Whitney‐Wilcoxon (MWW) U‐test. The chronological series reported the parameter values as recorded in the BreastWatch's Export File by six different experienced planners using the script for this specific treatment setting.

## 3 RESULTS

At the end of the testing phase, the gathered impressions of BreastWatch effectiveness were largely positive and it was found that the introduction of the BreastWatch script for the automatic analysis of EBBT plans in the planning practice can bring several advantages:

1. Quick assessment of plan *quality* leads to a faster planning process and more consistent results.
2. Automatic export of plan data allows to examine center‐specific results and to introduce new optimized dose‐constraints.
3. The Community‐Based Data Inspector tool lets users check plans against the Community Plan Library.
4. PSC signals minor planning pitfalls, which are often overlooked.

### 3.1 Quick assessment of plan *quality*

Compared to the Eclipse Clinical Goals interface, BreastWatch offers built‐in breast protocols, automatic protocol selection, and a more intuitive UI, tailor‐made for EBBT Plans. BreastWatch just takes some seconds to analyze an EBBT plan and shows at a glance, using traffic light colors, what is ok and what is not. This is also useful to quickly find out if a planning approach is going to easily fulfill protocol dose‐constraints or if something more complex is needed. Moreover, in addition to standard protocol dose‐constraints, BreastWatch offers extra dose‐constraints, an aspect particularly useful for protocols like ICR Fast Forward trial, which provides just a few ones.

### 3.2 Automatic export of plan data

BreastWatch offers a zero‐cost automatic export of plan data in the Export File. This feature allowed our center to build our own EBBT Community Plan Library useful to compute center‐specific optimized dose‐constraints and to perform statistical evaluation of dose metrics like OAR mean doses.

### 3.3 Community‐Based tool

Data Inspector allows the user to easily compare the dose values of a plan with Community plans. Given that the comparison is made by filtering data to select only proper compatible data, this feature allows the planner to critically judge his/her plan, compare it to what other planners are obtaining in similar situations, and find out weak points that can deserve further optimization.

**FIGURE 5:**
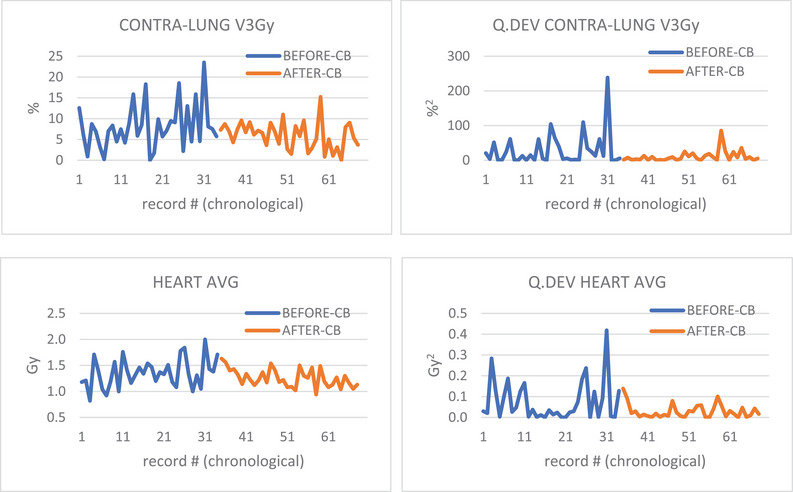
Chronological charts of Contra‐Lung V3Gy and Heart Average Dose and their quadratic deviations from the mean (Q.DEV), for RIGHT breast VMAT treatment with/without Boost and no DIBH with ICR Fast‐Forward Protocol. Orange curves refer to data for plans after the introduction of the Community‐Based Data Inspector tool in BreastWatch v.1.5. The Q.DEV of a function f(x) is defined as Q.DEV(x) = [f(x)— < f >]^2^ and highlights the absolute deviation from the average of the function < f >.

The Data Inspector tool can also improve consistency and robustness among different planners. Preliminary analysis of the BEFORE and AFTER datasets (see Figure 5) and MWW U‐test revealed that the AFTER dataset contains values somewhat improved with respect to the BEFORE dataset (one‐tail *p*‐value = 0.073 for both Heart Average Dose and Contra‐Lung V3Gy). MWW U‐test also reported a weakly significant improvement of quadratic deviation from the mean (Q.DEV), for Heart Average Dose (one‐tail *p*‐value = 0.074) and for Contra‐Lung V3Gy (one‐tail *p*‐value = 0.162) suggesting a trend in reduction of values dispersion hence a better planning homogeneity after CB introduction.

### 3.4 Plan sanity check

As detailed before, BreastWatch includes a basic PSC of EBBT plans. PSC features also a MU‐Sum Data Inspector (Figure 3), which compares the algebraic sum of the beams MUs (MU‐Sum) for the given plan with Community MU‐Sums for similar plans. This can be useful also to examine indirectly the complexity of IMRT or VMAT plans.

## 4 DISCUSSION

Modern EBBT planning is not only time‐consuming, but also requires new strategies to make planning to comply with international breast treatment protocols, while at the same time being able to adapt to patient conformation, center‐specific variations, new treatment techniques, and recent efforts to achieve challenging results like low‐dose cardiac sparing.^13^ The BreastWatch script was designed to automate and speed up the analysis of Eclipse EBBT plans, relying not only on standard EBBT protocol dose‐constraints but also on the feedback provided by the Community of local planners. This feedback is very important because, as evidenced before, standard protocol dose‐constraints are often generic, technique‐dependent, and not center‐specific.

A standard Varian Eclipse EBBT Plan, once planned and optimized, is visually analyzed to assess the plan *quality*. PTVs coverage, uniformity, and OARs dose‐constraints values can be manually collected using Eclipse DVH and Dose Statistics, but this requires a lot of time and it is error‐prone, so the preferred way for constraints analysis is to use Eclipse Clinical Goals, which specify constrained DVH values to be automatically calculated and reported. Unfortunately, in Eclipse v.13.5 the Clinical Goals user interface is linked to Eclipse Clinical Protocols, which are rather cumbersome to implement and not easy to use. Starting from Eclipse v.16, Eclipse Clinical Goals are much more convenient because they have been separated from Clinical Protocols, can be displayed on DVH and during IMRT/VMAT optimization, and can be saved in XML format for sharing. The BreastWatch script tool provides an alternative to Eclipse Clinical Goals, is tailor‐made for EBBT plans, and offers Community‐Based features. We find BreastWatch UI to be snappier and to provide more automatic processing (protocol to use, laterality, PTVs, and OARs auto‐matching) if compared to Eclipse Clinical Goals.

We have seen that BreastWatch's Community‐Based features allow users to build a center‐specific Plan Library and to use it to improve the treatment plans by looking at the comparison with similar Community plans. Compared to commercial KB AI systems like Varian Rapidplan,^7^, ^14^, ^15^ evidently the BreastWatch CB approach is rather basic: it makes use of a Plan Library *tout court* without any regularization and machine/deep learning algorithms, it considers discrete DVH values, not the full DVH curves, it does not consider any spatial information about patient and structures. However, we should consider that: (i) it's zero‐cost, so just analyze your plans and save to build your own EBBT Plan Library, (ii) it does not require the construction of EBBT DL models (and you need one for each separate protocol variation and breast laterality), and (iii) it automatically updates plan after plan. In the CB approach, the Community Plan Library provides a sort of feedback to planners, so we expected a positive effect on EBBT plan optimization. In effect, we have shown in our preliminary analysis that the introduction of the CB approach shows a trend (*p*‐value = 0.07) in pushing some EBBT plan *quality* metrics a little further in the improvement direction. This is also quite evident by looking at the graphs of Figure 5 for Contralateral Lung V3Gy, Heart Average Dose, and their Q.DEVs: the AFTER group seems to contain more consistent results with no evident outliers like the #31 record in the BEFORE group.

## 5 CONCLUSION

We think the BreastWatch script tool to be a nice addition for Eclipse EBBT planning, offering a simple but effective approach to automation of EBBT plan analysis and improvement. The script's evaluation methodology is based on two pillars: breast treatment protocols, which offer validated standard dose‐constraints, and Community‐Based data, which introduce the user‐base center‐specific experience.

BreastWatch has been extensively tested with more than 800 EBBT plans by six experienced planners and the gathered impressions of its usage in terms of effectiveness of plan evaluation have been largely positive. While clearly playing in a different league compared to KB ML/DL AI algorithms, BreastWatch's Community‐Based approach can be a valid paradigm to improve EBBT plans one step beyond standardized breast treatment protocols relying on generic dose‐constraints. In fact, our preliminary analysis of a specific subgroup of 68 plans evidenced for some dosimetric results a trend (*p*‐value = 0.07) in the improvement direction triggered by the usage of Community‐Based tools.

The BreastWatch v.1.6.5.0 plugin script for Varian Eclipse v.13.5 (v.1.7.5.0 for Varian Eclipse v.18) is freely available on request from the corresponding author. Forthcoming developments of the script will target Eclipse v.18 and include: improved support of bilateral treatments, enhanced Community data inspection with user‐driven filtering, secondary traffic lights coloring system, introduction of an overall CB‐SCORE, weighed scoring systems, text‐based protocols to allow users to create/edit EBBT protocols, complexity analysis of EBBT VMAT beams.

## AUTHOR CONTRIBUTIONS

Stefano Agostinelli designed and developed the BreastWatch plugin, including the Community‐Based scoring system, carried out data analysis, and wrote the original and revised manuscripts. Daniele Zefiro contributed with some initial conception. All the authors contributed with data collection and beta testing. Gladys Blandino and Alessandra Fozza also provided clinical advice.

## CONFLICT OF INTEREST STATEMENT

The authors declare no conflicts of interest.

## Data Availability

Chronological series data used for the preliminary analysis of the Community‐Based approach can be requested for academic purposes from the corresponding author.
